# The Regulatory Effect of Coaggregation Between *Fusobacterium nucleatum* and *Streptococcus gordonii* on the Synergistic Virulence to Human Gingival Epithelial Cells

**DOI:** 10.3389/fcimb.2022.879423

**Published:** 2022-04-29

**Authors:** Ruiqi Yang, Tingjun Liu, Chunfeng Pang, Yanling Cai, Zhengmei Lin, Lihong Guo, Xi Wei

**Affiliations:** ^1^ Hospital of Stomatology, Guanghua School of Stomatology, Sun Yat-sen University, Guangzhou, China; ^2^ Guangdong Provincial Key Laboratory of Stomatology, Sun Yat-sen University, Guangzhou, China

**Keywords:** coaggregation, *Fusobacterium nucleatum*, *Streptococcus gordonii*, synergistic virulence, human gingival epithelial cells

## Abstract

In subgingival plaque biofilms, *Fusobacterium nucleatum* is closely related to the occurrence and development of periodontitis. *Streptococcus gordonii*, as an accessory pathogen, can coaggregate with periodontal pathogens, facilitating the subgingival colonization of periodontal pathogens. Studies have shown that *F. nucleatum* can coaggregate with *S. gordonii* and colonize the subgingival plaque. However, most studies have focused on monocultures or coinfection of species and the potential impact of coaggregation between the two species on periodontal interactions to human gingival epithelial cells (hGECs) remains poorly understood. The present study explored the effect of coaggregation between *F. nucleatum* and *S. gordonii* on subgingival synergistic virulence to hGECs. The results showed that coaggregation inhibited the adhesion and invasion of *F. nucleatum* to hGECs compared with that in the *F. nucleatum* monoculture and coinfection group. Coaggregation and coinfection with *F. nucleatum* both enhanced *S. gordonii* adhesion to hGECs, but neither of the two groups affected *S. gordonii* invasion to hGECs compared with *S. gordonii* monoculture. The gene expression levels of *TLR2* and *TLR4* in hGECs in the coaggregation group were higher than those in the monoculture groups but lower than those in the coinfection group. Compared with coinfection, the coaggregation inhibited apoptosis of hGECs and promoted the secretion of the proinflammatory cytokines TNF-α and IL-6 by hGECs, showed a synergistic inflammatory effect, while coaggregation inhibited the secretion of the anti-inflammatory cytokine TGF-β1. Coaggregation enhanced the phosphorylation of p65, p38, and JNK proteins and therefore activated the NF-κB and MAPK signaling pathways. Pretreatment with a pathway antagonist/inhibitor decreased the phosphorylation levels of proteins and the secretion of TNF-α and IL-6. In conclusion, coaggregation inhibited the adhesion and invasion of *F. nucleatum* to hGECs. However, it enhanced the adhesion of *S. gordonii* to hGECs. Compared with coinfection, coaggregation inhibited the apoptosis of hGECs. The coaggregation coordinately promoted the secretion of TNF-α and IL-6 by hGECs through the TLR/NF-κB and TLR/MAPK signaling pathways while inhibiting the secretion of TGF-β1, thus aggravating the inflammatory response of hGECs.

## Introduction

The oral microbiome is comprised of more than 700 prevalent taxa at the species level (Dewhirst et al., 2010; Gao et al., 2018). The physical and metabolic interactions between bacteria, as well as bacteria and their hosts, promote the dynamic development of microbial communities and form dental plaque biofilms. Periodontitis is a common oral disease in which dental plaque biofilms are the main pathogenic factor (Frencken et al., 2017; Peres et al., 2019). In the process of dental plaque formation, different types of bacterial species recognize and bind to each other through coaggregation (Kolenbrander et al., 2010; Guo et al., 2014). The gram-negative bacterium, *Fusobacterium nucleatum*, is closely related to the occurrence and development of periodontitis, which could coaggregate with early and late colonizers (Kolenbrander et al., 2010; Okuda et al., 2012; Park et al., 2017; Wu et al., 2021). *Streptococcus gordonii* is commonly considered an early colonizer in the formation of dental plaque biofilms (Jakubovics and Kolenbrander, 2010; Nobbs et al., 2011; Jakubovics et al., 2021). With accumulating evidence showing that *S. gordonii* can coaggregate with periodontal pathogens, it has been newly recognized as an accessory pathogen for facilitating the subgingival colonization of periodontal pathogens (Daep et al., 2011; Hendrickson et al., 2017; Kuboniwa et al., 2017; Brown et al., 2018). Studies have shown that *F. nucleatum* can adhere to *S. gordonii* by the outer membrane proteins RadD and CmpA, which help *F. nucleatum* colonize the subgingival plaque (Kaplan et al., 2009; Lima et al., 2017).

The first defense barrier of periodontal tissue against microorganisms is gingival epithelial cells (GECs), which not only form an attachment to the tooth surface, but also form a physical and chemical barrier against infection (Kantrong et al., 2019). GECs can bind to bacteria through special receptors on the cell surface to release antimicrobial peptides such as human β-defensins (hBDs), cytokines, or proteases to resist the invasion of external risk factors and maintain epithelial microecological balance (Handfield et al., 2008; Lee and Yilmaz, 2021). As an opportunistic pathogen, *F. nucleatum* can not only adhere to and invade GECs (Han et al., 2000; Gursoy et al., 2008; Stathopoulou et al., 2010; Jung et al., 2017; Hung et al., 2018), but also promote the invasion of the nonperiodontal pathogen *Streptococcus cristatus* into GECs (Edwards et al., 2006). This suggests that in the subgingival environment, *F. nucleatum*, which is located in the same ecological locus as *S. gordonii*, may also influence the adhesion or invasion of *S. gordonii* into GECs.

Studies have shown that compared with *S. gordonii*, *Porphyromonas gingivalis*, and *Aggregatibacter actinomycetemcomitans*, *F. nucleatum* can effectively induce the inflammatory response of GECs and trigger high levels of interleukin (IL)-1β, IL-6, and IL-8, while *S. gordonii* shows the lowest ability to induce inflammation (Stathopoulou et al., 2010; Peyyala et al., 2012). Expression microarrays revealed that the biological pathways in GECs significantly impacted by *F. nucleatum* and *S. gordonii* included toll-like receptors (TLRs) and mitogen-activated protein kinase (MAPK) signaling pathways (Hasegawa et al., 2007). TLRs are innate immune pattern recognition receptors (PRRs) that can identify the proteins, nucleic acids, lipids of pathogenic microorganisms, and intermediate products and metabolites synthesized in the reaction process, such as the lipopolysaccharide (LPS) of gram-negative bacteria (Kantrong et al., 2019) and the lipoteichoic acid (LTA) of the cell wall of gram-positive bacteria (Saito et al., 2020). The downstream NF-κB and MAPK signaling pathways could be activated through MyD88-dependent pathways, inducing the expression of proinflammatory cytokines (IL-1β, IL-6, IL-8, tumor necrosis factor [TNF]-α) and anti-inflammatory cytokines (IL-10, transforming growth factor [TGF]-β1), which play an important role in inflammation, immune regulation, cell survival, and proliferation (Tartey and Takeuchi, 2017).

Previous studies explored the inflammatory effect of bacteria on GECs in monoculture or coinfection states. Coinfection is only a physical mixture of bacteria that cannot truly reflect the biological functions of bacteria in the flora. Interspecies physical attachment initiates signal transduction cascades that trigger important physical changes in partner species, which could not be observed by monospecies or coinfected species experiments. There is now strong evidence that cell-cell interactions could lead to phenotypic adaptations that affect physiological and pathological functions, such as adhesion, cooperation in substrate utilization, environmental adaptation, and virulence (Jakubovics et al., 2008a; Jakubovics et al., 2008b; Ramsey et al., 2011; Meuric et al., 2013). In recent years, RNA-Seq has been gradually applied to the analysis of transcriptional regulation stimulated by interactions between bacteria. The transcriptional responses of *S. gordonii* and *F. nucleatum* subsp. *nucleatum* to coaggregation had been reported (Mutha et al., 2018). Among the five subspecies of *F. nucleatum*, subsp. *nucleatum* and *polymorphum* are both associated with apical periodontitis and periodontitis (Han, 2015). But *F. nucleatum* subsp. *polymorphum* showed the greatest ability to increase phagocytic capacity of neutrophils and to block superoxide generation (Kurgan et al., 2017). Our previous study, for the first time, reported that coaggregation between *F. nucleatum* subsp. *polymorphum*, and *S. gordonii* altered bacterial transcriptional profiling and attenuated the immune responses of macrophages (Liu et al., 2021), which may provide some insights into the present study.

In a subgingival plaque, *F. nucleatum* and *S. gordonii* coexist in a limited ecological site through coaggregation. However, it is still unclear how coaggregation between *F. nucleatum* and *S. gordonii* influences the subgingival synergistic virulence to GECs. This study built coaggregation model of *F. nucleatum* subsp. *polymorphum* and *S. gordonii* to explore the effects of coaggregation on subgingival synergistic virulence to hGECs and analyze the relevant mechanisms. We aimed to deepen the understanding of coaggregation regulation between *F. nucleatum* and accessory pathogen, providing a new experimental basis for the inhibition of dental plaque biofilm formation and the prevention or treatment of periodontal disease.

## Materials and Methods

### Bacterial Strains and Growth Conditions

*F. nucleatum* subsp. *polymorphum* ATCC 10953 was grown in brain heart infusion (BHI) broth (Difco, USA) supplemented with 5 μg/ml hemin (Sigma-Aldrich, USA), 1 μg/ml vitamin K (Sigma-Aldrich, USA), and 0.5% yeast extract (Difco, USA). *S. gordonii* DL1 was grown in BHI broth. Both bacterial strains were grown under anaerobic conditions (N_2_ 90%, CO_2_ 5%, H_2_ 5%) at 37°C.

### Coaggregation of *F. nucleatum* subsp. *polymorphum* and *S. gordonii*

Coaggregation assays were performed in modified coaggregation buffer (CAB) containing 150 mM NaCl, 1 mM Tris HCl pH 8, 0.1 mM CaCl_2_, and 0.1 mM MgCl_2_ as previously described (Kaplan et al., 2009; Kaplan et al., 2014; Lima et al., 2017). The bacterial cells were collected at the late exponential phase of growth. The optical density at 600nm (OD_600nm_) of *F. nucleatum* subsp. *polymorphum* was measured to be 0.80 (~10^9^ CFU/mL), and the OD_600nm_ of *S. gordonii* was around 0.65 (~10^9^ CFU/mL). The colony-forming units (CFUs) of bacteria was quantified by incubating *F. nucleatum* subsp. *polymorphum* on 5% sheep blood agar plates and incubating *S. gordonii* on BHI agar plates in serial dilutions under anaerobic conditions. Bacterial cells were cleaned and resuspended in CAB to a final concentration of ~2×10^9^ CFU/mL. Equal numbers of bacterial cells from each species were added together and vortexed for 10 seconds in a new reaction tube. The suspensions were settled at room temperature for 10 min to allow the bacteria to coaggregate with each other. The reaction tube was centrifuged at low speed (100×g) for 1 min to pellet coaggregated bacterial cells while leaving the nonaggregated cells in the supernatant. The supernatant was collected carefully for OD_600nm_ measurement. The coaggregation index (C.I.) was calculated as follows (Kaplan et al., 2009; Kaplan et al., 2014): C.I. = (OD_600nm_(Fnp) + OD_600nm_ (Sg)-OD_600nm_ (Fnp-Sg))/[OD_600nm_ (Fnp)+OD_600nm_ (Sg)]. In this formula, OD_600nm_(Fnp) and OD_600nm_ (Sg) were the optical density of *F. nucleatum* subsp. *polymorphum* and *S. gordonii* respectively, while OD_600nm_ (Fnp-Sg) was the optical density of the supernatant after coaggregation. Because saliva is the common coaggregation buffer in the oral cavity, the coaggregation index of *F. nucleatum* subsp. *polymorphum* and *S. gordonii* in different concentrations of artificial saliva (Phygene, China) was also calculated. The coaggregation and autoaggregation of the two bacterial species in CAB at different time points were also evaluated and observed with phase contrast microscopy. The autoaggregation index was calculated as follows (Nagaoka et al., 2008; Karched et al., 2015; Toh et al., 2019): (OD_600nm_ (time zero value)- OD_600nm_ (sample value))/(OD_600nm_ (time zero value).

### Confocal Laser Scanning Microscopy Identification of Coaggregation of *F. nucleatum* subsp. *polymorphum* and *S. gordonii*

*F. nucleatum* subsp. *polymorphum* and *S. gordonii* were cultured to the late-exponential phase. Bacterial cells were washed three times and resuspended in sterile PBS. For visualization, *F. nucleatum* subsp. *polymorphum* was stained green with 5-(and-6)-carboxyfluorescein succinimidyl ester (CFSE) (Thermo Fisher, USA), while *S. gordonii* was stained red with hexidium iodide (Thermo Fisher, USA) according to the manufacturer’s instructions. Samples were incubated for 15 min in darkness at room temperature. Fluorescently stained bacteria were washed three times with sterile PBS and resuspended in CAB. The coaggregated *F. nucleatum* subsp. *polymorphum* and *S. gordonii* (Fnp-Sg) were obtained as described above. Coculture of the two species (Fnp+Sg) in PBS, where they did not coaggregate with each other but only mixed physically, were used as controls. After coaggregation reactions, 10 μL of coaggregated Fnp-Sg was transferred to a glass slide and covered with a cover glass. The coaggregation and coculture samples were visualized by an Olympus confocal microscope (FV3000, Olympus, Japan) using excitation (Ex) at 492 nm and emission (Em) at 517 nm for CFSE and Ex/Em = 518 nm/600 nm for hexidium iodide.

### Culture and Infection of Human Gingival Epithelial Cells *In Vitro*

Human gingival epithelial cells (hGECs) were obtained from the American Type Culture Collection (ATCC CRL-3397) and incubated in DMEM containing 10% fetal bovine serum (FBS) (Gibco, USA) at 37°C in the presence of 5% CO_2_ (Huang et al., 2020). Cells were seeded at 3.5 × 10^5^ cells per well in 6-well cell culture plates (Corning, USA). hGECs were infected with *F. nucleatum* subsp. *polymorphum* monoculture (Fnp), *S. gordonii* monoculture (Sg), coinfection of *F. nucleatum* subsp. *polymorphum* and *S. gordonii* (Fnp+Sg), and coaggregation of *F. nucleatum* subsp. *polymorphum* and *S. gordonii* (Fnp-Sg) at an MOI of 100, respectively. The coinfection of *F. nucleatum* subsp. *polymorphum* and *S. gordonii* (Fnp+Sg) was only a physical mixture of bacteria in PBS where they did not coaggregate with each other. To ensure the number of bacterial cells in coaggregates was similar with monocultures, the coaggregates were resuspended in PBS, vigorously vortexed and disrupted until no visible pellet existed with validation under a microscope (Liu et al., 2021). The CFU of the coaggregates were determined by incubating the resuspension solution on 5% sheep blood agar plates and BHI agar plates in serial dilutions. The volumes of bacterial cells used in the coaggregation group were adjusted to ensure the number of bacterial cells in coaggregates were similar with monoculture groups. After incubation at 37°C in 5% CO_2_ for 4 hours, the culture medium containing bacteria was removed, and the bacteria were washed with PBS three times to remove planktonic bacteria. Cells in each well were added to 2 mL of DMEM containing 10% FBS, 200 μg/mL metronidazole (Solarbio, China) and 300 μg/mL of gentamicin (Solarbio, China) and incubated at 37°C in 5% CO_2_ for 60 min, 90 min, and 120 min to test the antibiotic treatment time for completely killing of extracellular bacteria in all groups. In detail, hGECs infected with Fnp, Sg, Fnp+Sg, and Fnp-Sg of the same antibiotic treatment time were digested and mixed together. The effect of killing extracellular bacteria was confirmed by incubating the digested cells mixture on a plate containing 10% sterile sheep’s blood at 37°C with 90% N_2_ + 5% CO_2_ + 5% H_2_ for 2-3 days. If bacterial colonies grew on the plate, it meant not all groups achieved a complete killing of extracellular bacteria. After killing the extracellular bacteria, cells were washed with PBS three times and incubated at 37°C in 5% CO_2_ for different time points. The experiment was performed three times.

### Confocal Laser Scanning Microscopy (CLSM) Evaluation of hGEC Infection by *F. nucleatum* subsp. *polymorphum* and *S. gordonii In Vitro*

To examine bacterial infection, CFSE-labeled *F. nucleatum* subsp. *polymorphum* and *S. gordonii* were cocultivated with hGECs for 4 hours on cell slides. After infection, hGECs were washed 3 times with PBS to remove planktonic bacteria. The cells were fixed with 4% paraformaldehyde for 15 min and treated with 0.1% Triton X-100 (Beyotime, China) for 10 min. The cytoskeleton was stained with phalloidin (Thermo Fisher, USA) for 30 min, and the nucleus was stained with DAPI (ZSGB-BIO, China). All CLSM images were obtained by an Olympus confocal microscope using Ex/Em = 492 nm/517 nm for CFSE, Ex/Em = 540 nm/565 nm for phalloidin and Ex/Em = 340 nm/488 nm for DAPI.

### Adhesion and Invasion Assay

hGECs were infected with Fnp, Sg, Fnp+Sg, and Fnp-Sg at a MOI of 100. After 4 hours of infection, the cells were washed 3 times with PBS to remove the planktonic bacteria and lysed in sterile water for 90 min to release intracellular bacteria. The total number of *F. nucleatum* subsp. *polymorphum* and *S. gordonii* adhering to and invading hGECs was counted by serial dilution and plating on BHI agar supplemented with yeast extract, hemin, and vitamin K. The agar plates were incubated anaerobically at 37°C with 90% N_2_ + 5% CO_2_ + 5% H_2_ for 2-3 days. For the invasion assay, after 4 hours of infection, the cells were washed 3 times with PBS to remove planktonic bacteria and treated with fresh DMEM supplemented with 10% FBS, 200 μg/mL metronidazole, and 300 μg/mL gentamicin for 120 min to kill extracellular bacteria. Cells were lysed in sterile water for 90 min and the number of intracellular bacteria was determined by serial dilution and plating as described above.

### Cell Viability of hGECs Infected by *F. nucleatum* subsp. *polymorphum* and *S. gordonii*

hGECs were inoculated into a 96-well plate (200 μL/well) at a density of 1.0×10^4^ cells per well. Fnp, Sg, Fnp+Sg and Fnp-Sg were added to cells at an MOI of 100. hGECs without any bacterial stimuli were used as the blank control group (control). After 4 hours, samples were treated with DMEM supplemented with 10% FBS, 200 μg/mL metronidazole, and 300 μg/mL gentamicin for 120 min and fresh DMEM supplemented with 10% FBS was added. The proliferation activity of hGECs was determined by the Cell Counting Kit-8 (CCK8 kit, Dojindo, Japan). After the addition of 10 μL of CCK8 solution to each well, the plate was incubated at 37°C in 5% CO_2_ for 1-4 h. The absorbance at 450 nm (OD_450nm_) was detected by a microplate reader. The effect of antibiotics alone on the proliferation activity of hGECs was also determined.

### Cell Apoptosis of hGECs Infected by *F. nucleatum* subsp. *polymorphum* and *S. gordonii*

hGECs were inoculated into a 6-well plate at 3.5×10^5^ cells per well. Bacterial stimuli were added as described above and hGECs without any bacterial stimuli were used as blank control. After antibiotic treatment, cells were cultured at 37°C in 5% CO_2_ and digested with trypsin without EDTA at different time points. Cells were washed with PBS twice and collected in flow cytometry tubes with 1~5×10^5^ cells by centrifugation at 1000 rpm for 5 min. An Annexin V-FITC Apoptosis Kit (BD, USA) was used to detect the apoptosis of hGECs according to the manufacturer’s instructions. After 500 μL of binding buffer was used to resuspend the cells, 5 μL of Annexin V-FITC was added and mixed gently. Samples were placed on ice for 15 min, mixed with 5 μL of propodium iodide (PI) and then detected by Beckman Coulter CytoFLEX immediately using Ex/Em = 488 nm/530 nm. Cells without Annexin V-FITC and PI were used as negative controls.

### RT–qPCR of the mRNA Expression Levels of *TLR2* and *TLR4* in hGECs Infected by *F. nucleatum* subsp. *polymorphum* and *S. gordonii*

The expression levels of *TLR2* and *TLR4* in hGECs were determined by quantitative reverse transcription PCR (RT–qPCR). Total RNA was isolated from hGECs using RNAzol according to the manufacturer’s protocol (Sigma-Aldrich, USA). The concentrations of RNA samples were determined by a NanoDrop 2000C Spectrophotometer (Thermo Fisher, USA). cDNA was synthesized using PrimeScript RT Master Mix (Takara, Japan). RT–qPCR analysis was performed in a 20-μL reaction mixture containing 10-μL of master mix (Hieff qPCR SYBR Green Master Mix, Yeasen) using a Light Cycler 480 (Roche Applied Science, Germany). The reaction product was quantified by the standard curve method. Levels of GAPDH mRNA served as internal controls. The primer sequences were as follows (F/R): TLR2 (ATCAGGCTTCTCTGTCTTGTG/TCTGTAGGTCACTGTTGCTAATG); TLR4 (GGAAGGAGCAGAATCAGGATATG/CTCCATTCACTCCACTAACCAC); and GAPDH (AATCCCATCACCATCTTCCAG/AAATGAGCCCCAGCCTTC).

### Cytokine Detection

The hGECs were stimulated with Fnp, Sg, Fnp+Sg and Fnp-Sg as described above. Cell-free supernatants were harvested and stored at -80°C for cytokine assays. Cytokine levels (TNF-α, IL-6, -8, -10 and TGF-β1) in the culture supernatants were measured by ELISA kits (Neobioscience, Shenzhen, China) according to the manufacturer’s instructions.

### The Activation of the NF-κB and MAPK Signaling Pathways in hGECs Infected With *F. nucleatum* subsp. *polymorphum* and *S. gordonii*

After removing the supernatant, the cells were washed twice with PBS at 4°C. RIPA lysis buffer (Beyotime, China) containing 1% protease inhibitor (Sigma–Aldrich, USA) and 1% serine protease inhibitor (Sigma–Aldrich, USA) was added for 30 min to lyse cells and extract proteins from each sample. The concentrations of total proteins were detected by a BCA protein assay kit (Beyotime, China) according to the manufacturer’s instructions. Protein samples were mixed with 5× loading buffer (ThermoFisher, USA) at a ratio of 4:1 and boiled at 99°C for 10 min. Samples were loaded and run on SDS–PAGE gels (CWBIO, China) and transferred onto PVDF membranes (Millipore, USA). Membranes were blocked with 2% skim milk (BD, USA) for 1 h at room temperature and then incubated with anti-IKKα, anti-IKKβ, anti-pIKKα/β, anti-p65, anti-pp65, anti-p38, anti-pp38, anti-SAPK/JNK, anti-pSAPK/JNK, and anti-GAPDH primary antibodies (Abcam, UK) overnight at 4°C. After primary incubation, blots were washed and incubated with secondary goat anti-rabbit or goat anti-mouse HRP (Abcam, UK) for 1 hour. Membranes were washed and exposed to chemiluminescent HRP substrate (Millipore, USA). Images were obtained using the GeneGnome XRQ system (Syngene, USA) and analyzed using ImageJ software.

### The Inhibition of the NF-κB and MAPK Signaling Pathways in hGECs Infected With *F. nucleatum* subsp. *polymorphum* and *S. gordonii*

Before bacterial stimuli, hGECs were pretreated the TLR2/4 signaling pathway antagonist OxPAPC (*In vivo*Gen, USA) at 30 μg/mL, 400 nM p38 inhibitor SB 239063 (MCE, USA), 2 μM JNK inhibitor SP600125 (MCE, USA), and 4 μM NF-κB signaling pathway inhibitor BAY 11-7082 (MCE, USA) for 1 h. hGECs treated without any inhibitors and bacterial stimuli were used as blank controls, hGECs treated with inhibitors but without any bacterial stimuli were used as negative controls, and hGECs treated with Fnp-Sg infection but without any inhibitors were used as positive controls. Total proteins were extracted at 24 h and 48 h after incubation. The phosphorylation of NF-κB pathway protein p65, MAPK pathway protein p38 and JNK was detected by western blot as described above. The supernatant was collected and used to detect the changes in the secretion of inflammatory cytokines by ELISA.

### Statistical Analysis

The bacterial counts data were log (10) transformed for subsequent analysis. All data were presented as the mean ± standard deviation and assessed for normality by Kolmogorov-Smirnov test. The results showed that the data fitted a normal distribution. Differences between two groups were analyzed by Student’s *t* tests. Differences in the quantitative data between multiple groups were evaluated by one-way ANOVA combined with Bonferroni’s *post hoc* test. *P* values less than 0.05 were designated as significant differences. Statistical analyses were conducted by SPSS Statistics v.20 software (IBM, Inc., Chicago, IL, USA) and GraphPad Prism 9 software (GraphPad Software, Inc., San Diego, CA, USA).

## Results

### The Coaggregation Between *F. nucleatum* subsp. *polymorphum* and *S. gordonii*

**Figure 1:**
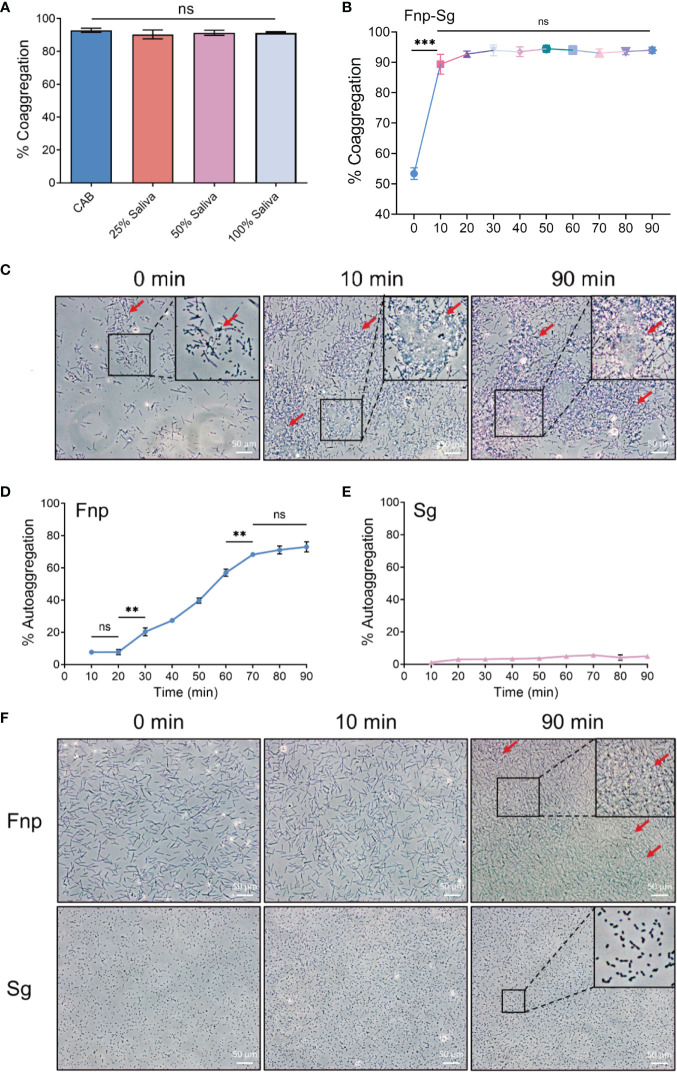
**(A)** Quantitative coaggregation assays between *F*. *nucleatum* subsp. *polymorphum* and *S. gordonii* in CAB and different concentrations of artificial saliva. **(B)** The stability of coaggregation between *F*. *nucleatum* subsp. *polymorphum* and *S. gordonii* in CAB in 90 min. **(C)** Phase contrast microscopy images of coaggregation between *F*. *nucleatum* subsp. *polymorphum* and *S. gordonii* in CAB at 0, 10 and 90 min (red arrows: coaggregates). **(D)** The autoaggregation of *F*. *nucleatum* subsp. *polymorphum* at 90 min. **(E)** The autoaggregation of *S. gordonii* at 90 min. **(F)** Phase contrast microscopy images of autoaggregation of *F*. *nucleatum* subsp. *polymorphum* and *S. gordonii* in CAB at 0, 10 and 90 min (red arrows: autoaggregates) (**p <0.01, ***p <0.001, ns: not statistically significant).

Studies have confirmed that different bacterial strains coaggregate adequately with each other in CAB (Kaplan et al., 2009; Kaplan et al., 2014). Saliva was also used as a coaggregation buffer, of which the composition was complex and included various enzymes, immunoglobulins, and mucins (Heller et al., 2017; Carpenter, 2020). In the present study, *F. nucleatum* subsp. *polymorphum* coaggregated strongly with *S. gordonii* in 10 min with large numbers of coaggregation clumps formed at the bottom of the centrifuge tube and a clear upper suspension. The coaggregation index (C.I.) was 89.370% ± 3.269% (mean ± standard deviation). There was no significant difference between coaggregation indices in CAB and different concentrations of artificial saliva (**Figure 1A**). The coaggregation was stable in 10-90 min with a range of coaggregation indices from 89.370% ± 3.269% to 94.450% ± 1.161% (**Figures 1B, C**). Thus, CAB was used in the present study to exclude the influence of saliva components on the results. Autoaggregation is the adhesion of bacteria of the same strain, which is common with oral bacteria (Khemaleelakul et al., 2006; Merritt et al., 2009). It is mediated by autoagglutinins which is related to surface proteins in general or related to carbohydrates, particularly exopolysaccharides in some cases (Trunk et al., 2018; Yakovlieva and Walvoort, 2020). Although *F. nucleatum* had been extensively studied on coaggregation, little was known about its autoaggregation. Previous studies showed the autoaggregation of *F. nucleatum* was strain-dependent and occurs *via* both saliva-dependent and -independent mechanisms (Merritt et al., 2009; Karched et al., 2015). There were few studies focused on the autoaggregation of *S. gordonii*. A previous study showed no autoaggregation of *S. gordonii* (Levin-Sparenberg et al., 2016), while another study showed an increased autoaggregation of *S. gordonii* depending on the concentration of composite resin containing surface reaction-type pre-reacted glass ionomer eluate used (Shimazu et al., 2016). However, the specific mechanisms of autoaggregation in *F. nucleatum* and *S. gordonii* still needed further investigations. In the present study, the autoaggregation of *F. nucleatum* subsp. *polymorphum* significantly increased at 20 min (**Figure 1D**) which was consistent with a previous study (Karched et al., 2015). We chose 10 min as the coaggregation time in the present study when there was little autoaggregation of *F. nucleatum* subsp. *polymorphum* and *S. gordonii* (**Figures 1E, F**).

**Figure 2:**
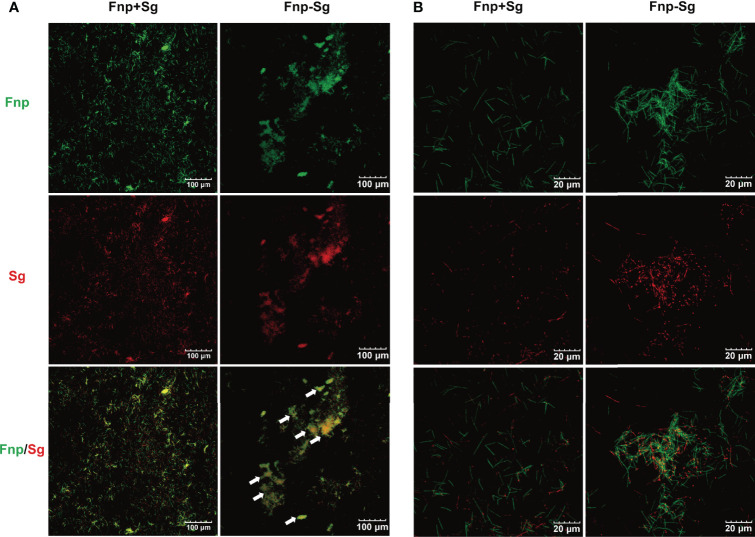
CLSM images of the coculture (Fnp+Sg) and coaggregation (Fnp-Sg) of *F*. *nucleatum* subsp. *polymorphum* (green) and *S. gordonii* (red) at low magnification **(A)** and high magnification **(B)**. The white arrows show the coaggregates.

CLSM images at low magnification showed coaggregation between *F. nucleatum* subsp. *polymorphum* and *S. gordonii* in the form of a large number of clumps (white arrows), while the coculture *F. nucleatum* subsp. *polymorphum* and *S. gordonii* were noncoaggregated and distributed separately (**Figure 2A**). Under high magnification, the coaggregation group showed that *F. nucleatum* subsp. *polymorphum* and *S. gordonii* cells adhered to each other tightly and were distributed quite evenly throughout coaggregates (**Figure 2B**).

### The Effect of Antibiotics on Killing Extracellular Bacteria and Cell Proliferation of hGECs

The results showed that the number of extracellular bacteria decreased with prolonged antibiotic treatment time (** p <0.01, *** p <0.001). After 120 min, no visible bacterial colonies grew, indicating the complete killing of extracellular bacteria among all the groups with bacterial stimuli (**Figure S1A**). The CCK-8 results showed that antibiotic treatment had no significant effect on the proliferation of hGECs (**Figure S1B**). Therefore, the extracellular bacteria were killed by antibiotic treatment for 120 min in the present study.

### The Infection of hGECs by *F. nucleatum* subsp. *polymorphum* and *S. gordonii*

**Figure 3:**
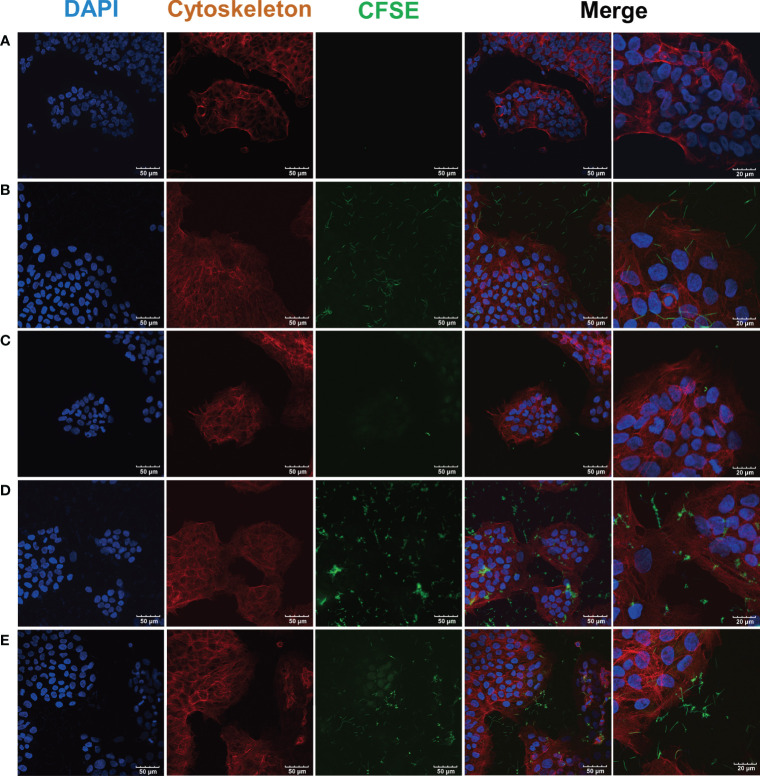
CLSM images of *F*. *nucleatum* subsp. *polymorphum* and *S. gordonii* infection of hGECs for 4 h *F. nucleatum* subsp. *polymorphum* and *S. gordonii* were stained green by CFSE. The cytoskeleton was stained red by phalloidin, and the nucleus was stained blue by DAPI. **(A)** Blank control group. **(B)** *F. nucleatum* subsp. *polymorphum* monoculture group (Fnp). **(C)** *S. gordonii* monoculture group (Sg). **(D)** *F. nucleatum* subsp. *polymorphum* and *S. gordonii* coinfection group (Fnp+Sg). **(E)** *F. nucleatum* subsp. *polymorphum* and *S. gordonii* coaggregation group (Fnp-Sg).

As the CLSM images showed, cell cytoskeleton was stained red with phalloidin and the nucleus was stained blue with DAPI (**Figure 3A**). There was a large number of *F. nucleatum* subsp. *polymorphum* infected- hGECs (**Figure 3B**), while *S. gordonii* hardly infected hGECs (**Figure 3C**). Compared with the coinfection group (**Figure 3D**), the number of *F. nucleatum* subsp. *polymorphum* and *S. gordonii* that infected hGECs seemed reduced in the coaggregation group (**Figure 3E**).

**Figure 4:**
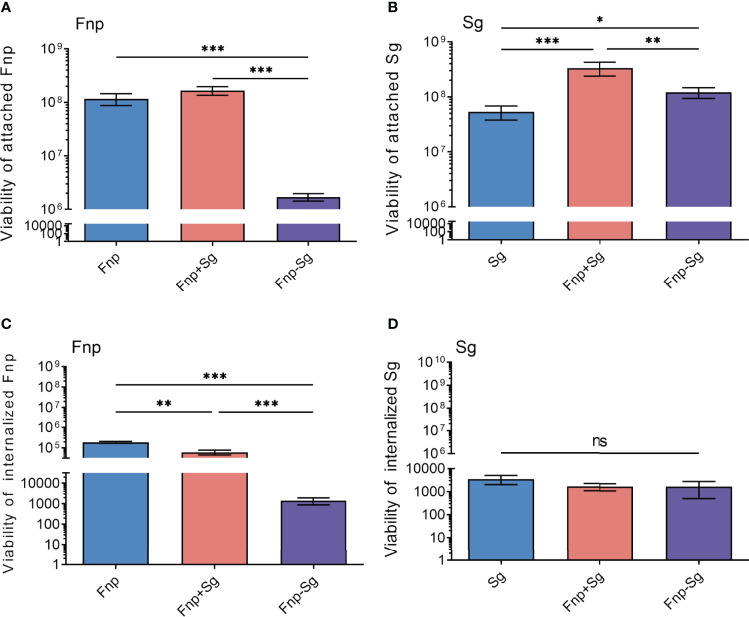
The numbers of attached *F*. *nucleatum* subsp. *polymorphum* **(A)** and *S. gordonii*** (B)**, internalized *F*. *nucleatum* subsp. *polymorphum* **(C)** and *S. gordonii* **(D)** to hGECs with an MOI of 100 after 4 h of infection (*p <0.05, **p <0.01, ***p <0.001, ns: not statistically significant).

To further explore the infection of *F. nucleatum* subsp. *polymorphum* and *S. gordonii* to hGECs, we quantified the bacteria that adhered to and invaded hGECs by serial dilution and plating, respectively. The results showed that coaggregation significantly inhibited *F. nucleatum* subsp. *polymorphum* adhesion and invasion of hGECs (** *p <0.01*, *** *p <0.001*) (**Figures 4A, C**). For *S. gordonii*, coaggregation and coinfection both enhanced the adhesion of hGECs, with the coinfection group showing a stronger effect than the coaggregation group (* *p <0.05*, ** *p <0.01*, *** *p <0.001*) (**Figure 4B**). Nevertheless, the invasion ability of *S. gordonii* in hGECs was weak among all groups and neither coinfection nor coaggregation influenced the invasion ability of *S. gordonii* (**Figure 4D**).

### The Effect of Coaggregation Between *F. nucleatum* subsp. *polymorphum* and *S. gordonii* on the Proliferation Activity and Apoptosis of hGECs

**Figure 5:**
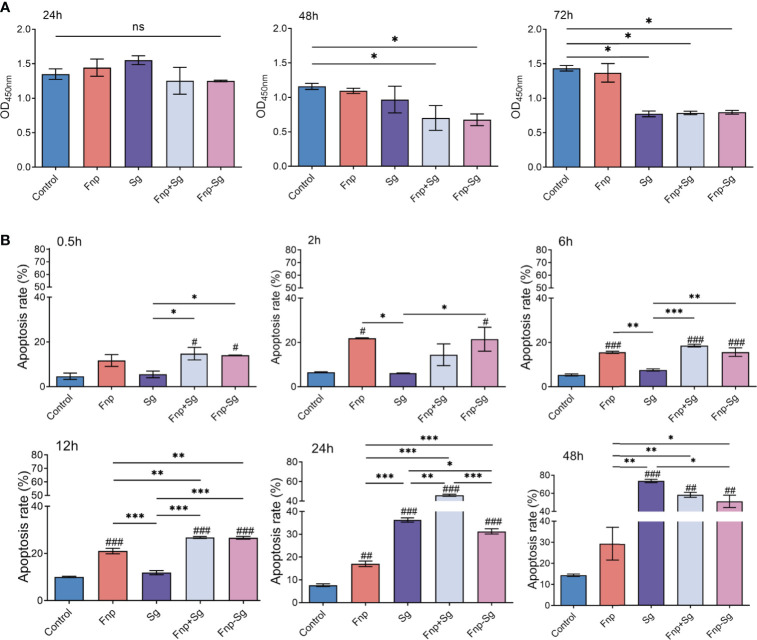
**(A)** The proliferation activity of hGECs infected by Fnp, Sg, Fnp+Sg and Fnp-Sg after 24, 48 and 72 h of infection (*p <0.05, ns: not statistically significant). **(B)** The apoptosis rate of hGECs infected with Fnp, Sg, Fnp+Sg and Fnp-Sg after various time points (*p < 0.05, **p < 0.01, ***p < 0.001; ^#^p < 0.05, ^##^p < 0.01, ^###^p < 0.001, compared with the control group).

The results showed that the monocultures, coinfection, and coaggregation of *F. nucleatum* subsp. *polymorphum* and *S. gordonii* had no significant effect on the proliferation activity of hGECs after 24 h of infection (**Figure 5A**). After 48 h of infection, the proliferation activity of hGECs was significantly reduced in both the coinfection and coaggregation groups with no significant difference between the two groups (* *p <0.05*). The monoculture of *S. gordonii* inhibited the proliferation activity of hGECs after 72 h of infection (* *p <0.05*), while *F. nucleatum* subsp. *polymorphum* monoculture had no significant influence on the proliferation activity of hGECs at various time points.

As shown in **Figure 5B**, *F. nucleatum* subsp. *polymorphum* showed a relatively weak ability to promote cell apoptosis after 2 h of infection and sustained through 24 h. After 12 h of infection, the coaggregation and coinfection of *F. nucleatum* subsp. *polymorphum* and *S. gordonii* significantly promoted hGECs apoptosis compared with the other groups (^###^ *p <0.001*, ** *p <0.01*, *** *p <0.001*, ^#^compared with the control group). There was no significant difference between the coinfection and coaggregation groups within 12 h of infection, however, the coinfection group significantly promoted cell apoptosis at 24 h compared with the coaggregation group (*** *p <0.001*). After 24 h of infection, the ability of *S. gordonii* to promote cell apoptosis was enhanced and was the strongest after 48 h. The images of flow cytometry were shown in **Figure S2**.

### The Effect of Coaggregation Between *F. nucleatum* subsp. *polymorphum* and *S. gordonii* on *TLR2* and *TLR4* mRNA Expression Levels in hGECs

**Figure 6:**
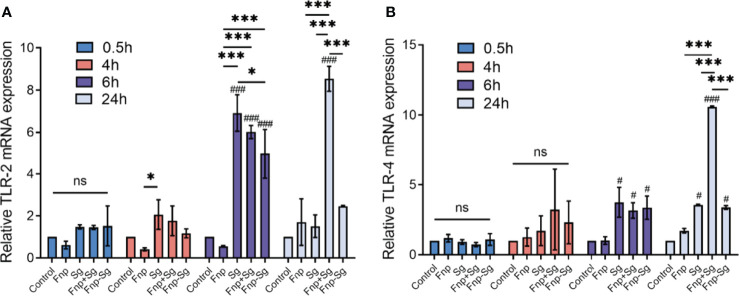
*TLR2* **(A)** and *TLR4* **(B)** mRNA expression levels in hGECs infected with Fnp, Sg, Fnp+Sg and Fnp-Sg (*p < *0.05*, ***p < *0.001*, ^#^compared with the control group, ^#^ *p* < *0.05*, ^###^ *p* < *0.001*, ns: not statistically significant).

RT–qPCR was used to detect the effect of coaggregation between *F. nucleatum* subsp. *polymorphum* and *S. gordonii* on the *TLR2* and *TLR4* mRNA expression levels in hGECs (**Figure 6**). The results showed that after 6 h of infection, the *TLR2* and *TLR4* mRNA expression levels were significantly increased in the *S. gordonii* monoculture, coinfection, and coaggregation groups compared with the control group (^#^ *p* < *0.05*, ^###^ *p* < *0.001*). After 24 h of infection, the *TLR2* and *TLR4* mRNA expression levels were significantly decreased in the coaggregation group compared with the coinfection group (***p <0.001). In the present study, *S. gordonii* monoculture increased both *TLR2* and *TLR4* mRNA expression levels in hGECs, while *F. nucleatum* subsp. *polymorphum* monoculture had no significant influence on the expression levels.

### The Effect of Coaggregation Between *F. nucleatum* subsp. *polymorphum* and *S. gordonii* on the Secretion of Inflammatory Cytokines by hGECs

**Figure 7:**
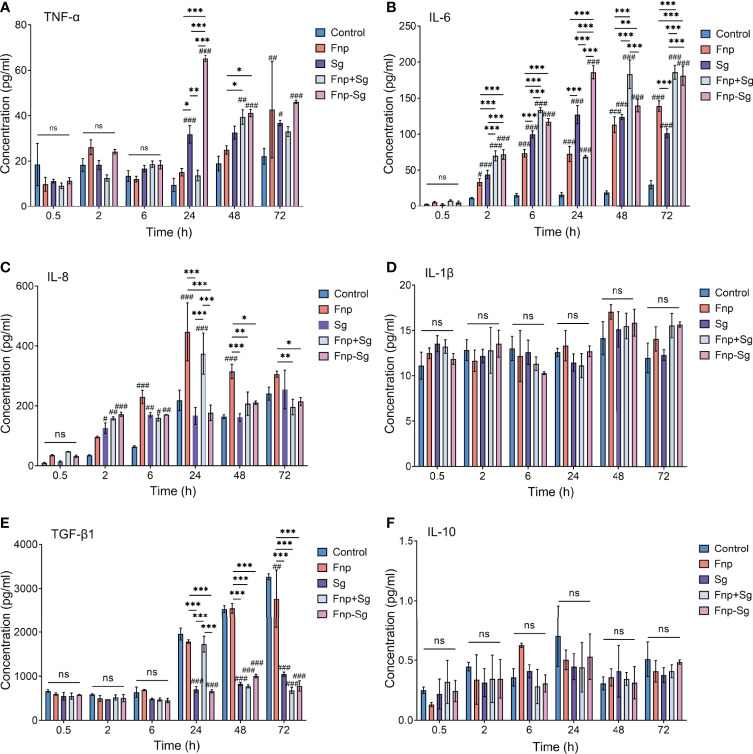
The production of TNF-α, IL-6, IL-8, IL-1β, TGF-β1 and IL-10 in hGECs infected with Fnp, Sg, Fnp+Sg and Fnp-Sg, as assessed by ELISA (*p < *0.05*, **p < *0.01*, ***p < *0.001*; ^#^ *p* < *0.05*, ^##^ *p* < *0.01*, ^###^ *p* < *0.001*, ^#^compared with the control group, ns: not statistically significant).

ELISA results showed that the secretion level of TNF-α by hGECs in the coaggregation group was significantly higher than that in other groups at 24 h of infection (***p <0.001, ^###^ *p <0.001*, ^#^compared with the control group), while no difference was found between groups at 0.5 h, 2 h, and 6 h. Afterwards, the secretion level of TNF-α in the coaggregation group decreased with no difference compared with the coinfection group (**Figure 7A**). The secretion level of IL-6 steadily reached the highest in the coaggregation group at 24 h of infection, showing a significantly higher level than that in other groups (***p <0.001, ^###^ *p <0.001*, ^#^compared with the control group). The secretion level of IL-6 in the coinfection group increased within 6 h and decreased at 24 h of infection. However, the secretion level of IL-6 in the coinfection group restored and was dramatically higher than that in the coaggregation group at 48 h of infection (**Figure 7B**). As for IL-8, *F. nucleatum* subsp. *polymorphum* monoculture and coinfection groups resulted in high secretion levels at 24 h of infection and then decreased. The coaggregation group did not significantly promote IL-8 secretion at various time points (**Figure 7C**). The secretion levels of TGF-β1 were higher in the control and *F. nucleatum* subsp. *polymorphum* monoculture groups than that in other groups at 48 h and 72 h of infection (***p <0.001, ^###^ *p <0.001*, ^#^compared with the control group). However, the secretion level of TGF-β1 in the coinfection group increased significantly at 24 h with no difference compared with the control and *F. nucleatum* subsp. *polymorphum* monoculture groups and decreased afterwards (**Figure 7E**). No significant difference was found in the secretion of IL-1β and IL-10 among the groups (**Figures 7D, F**).

### Activation of the NF-κB and MAPK Signaling Pathways in hGECs Infected by Coaggregation Between *F. nucleatum* subsp. *polymorphum* and *S. gordonii*

**Figure 8:**
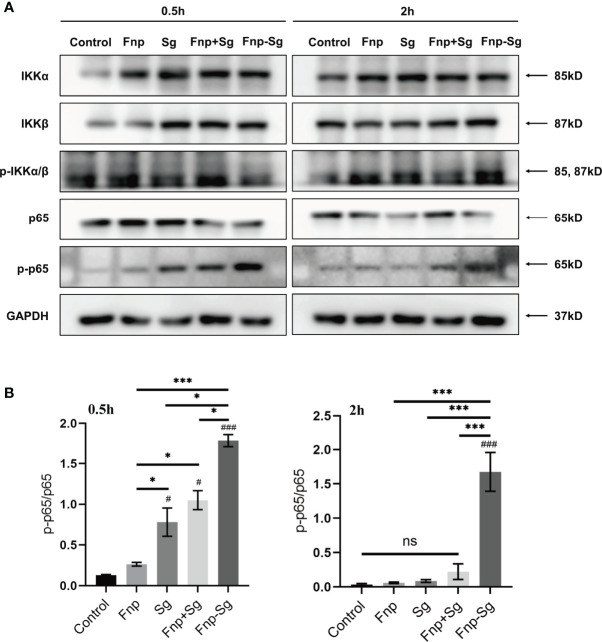
The phosphorylation levels **(A)** and semiquantitative analysis **(B)** of proteins in NF-κB signaling pathways in hGECs infected with Fnp, Sg, Fnp+Sg and Fnp-Sg (*p < *0.05*, ***p < *0.001*; *^#^p* < *0.05*, *^###^p* < *0.001*, *^#^* compared with the control group; ns: not statistically significant).

**Figure 9:**
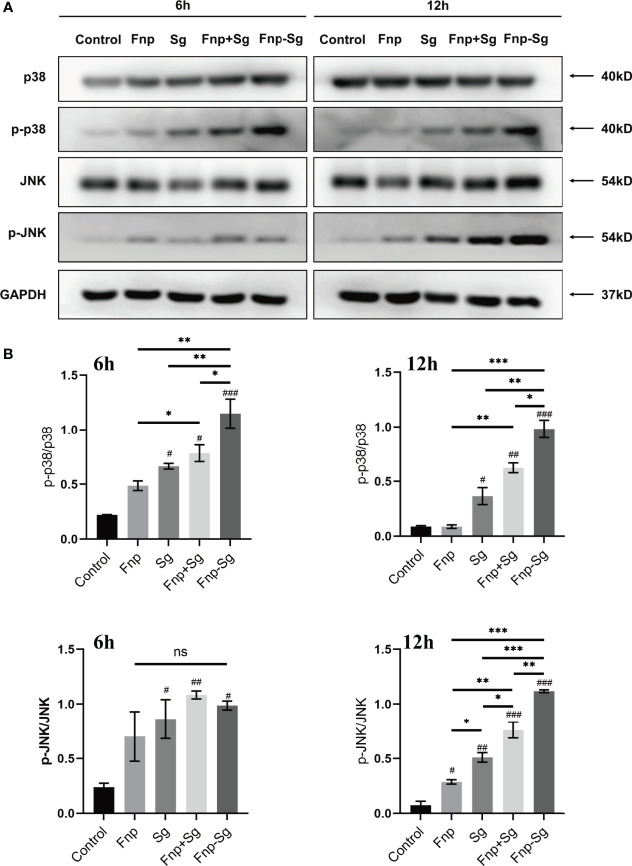
The phosphorylation levels **(A)** and semiquantitative analysis **(B)** of proteins in MAPK signaling pathways in hGECs infected with Fnp, Sg, Fnp+Sg and Fnp-Sg (*p < *0.05*, **p < *0.01*, ***p < *0.001*; *^#^p* < *0.05*, *^##^p* < *0.01*, *^###^p* < *0.001*, *^#^* compared with the control group; ns: not statistically significant).

The western blot and semiquantitative analysis results showed that the phosphorylation level of p65 (p-p65) protein in the coaggregation group was higher than that of the other groups after 0.5 h and 2 h of infection (* p <0.05, ** p <0.01, *** p <0.001, ^#^p <0.05, ^###^p <0.001, ^#^compared with the control group) (**Figure 8**). Moreover, the phosphorylation level of p38 (p-p38) protein in the coaggregation group was higher than that of the other groups after 6 h and 12 h of infection (* p <0.05, ** p <0.01, *** p <0.001, ^#^p <0.05, ^##^p <0.01, ^###^p <0.001, ^#^compared with the control group) (**Figure 9**). After 12 h of infection, the phosphorylation level of JNK (p-JNK) protein in the coaggregation group was higher than that in the other groups (* p <0.05, ** p <0.01, *** p <0.001, ^#^p <0.05, ^##^p <0.01, ^###^p <0.001, ^#^compared with the control group) (**Figure 9**).

### The Regulation of the NF-κB and MAPK Signaling Pathways in hGECs by Coaggregation Between *F. nucleatum* subsp. *polymorphum* and *S. gordonii*

**Figure 10:**
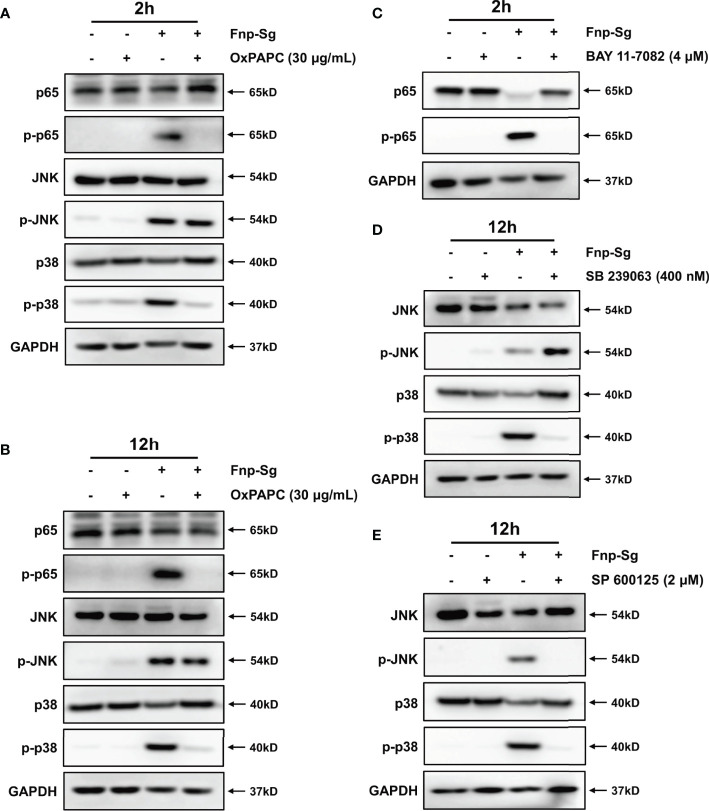
The regulations of the NF-κB and MAPK signaling pathways in hGECs by coaggregation between *F*. *nucleatum subsp. polymorphum* and *S. gordonii* by western blot. hGECs were pretreated with the TLR2/4 antagonist OxPAPC for 2 h **(A)** and 12 h **(B)**, the NF-κB inhibitor BAY 11-7082 **(B)** for 2 h **(C)**, the p38 MAPK inhibitor SB 239063 for 12 h **(D)** and the JNK MAPK inhibitor SP 600125 for 12 h **(E)**.

By pretreatment of hGECs with the TLR2/4 antagonist OxPAPC for 1 h, the protein expression of p-p65, p-p38, and p-JNK in the coaggregation group was decreased (**Figures 10A, B**). After pretreatment with the NF-κB inhibitor BAY 11-7082, the protein expression of p-p65 decreased significantly (**Figure 10C**). Pretreatment with the p38 MAPK inhibitor SB 239063 decreased the p-p38 protein level; thus, the p-JNK protein level showed a compensatory increase (**Figure 10D**). Pretreatment with the JNK MAPK inhibitor SP 600125 significantly decreased the p-JNK protein level. Because the targets of the inhibitor SP 600125 also included the upstream kinases MKK3, MKK4, and MKK6 in the p38 MAPK signaling pathway, the p-p38 protein level was decreased significantly (**Figure 10E**).

**Figure 11:**
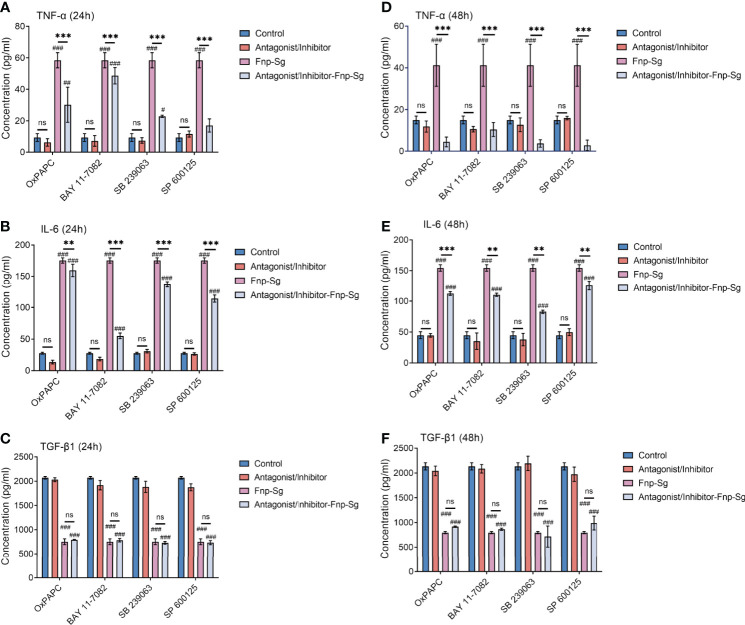
The secretion levels of inflammatory cytokines by hGECs pretreated with antagonist or inhibitors at 24 h **(A–C)** and 48 h of infection **(D–F)** (**p < *0.01*, ***p < *0.001*, ^##^ *p* < *0.01*, ^###^ *p* < *0.001*, ^#^compared with the control group, ns: not statistically significant).

The changes in the secretion of inflammatory cytokines by hGECs were also evaluated by ELISA. By pretreatment of hGECs with antagonist or inhibitors, the levels of TNF-α and IL-6 decreased in the coaggregation group after 24 h and 48 h of infection (** *p* < *0.01*, *** *p* < *0.001*) (**Figures 11A, B, D, E**), but no significant difference was detected in the secretion of TGF-β1 (**Figures 11C, F**).

## Discussions

Coaggregation with early colonizers is important for the colonization of *F. nucleatum* in the oral flora (Guo et al., 2014). Although *S. gordonii* are generally considered early colonizers and commensal organisms, increasing evidence shows that they are becoming recognized as important associated pathogens during the development of periodontal disease (Croft et al., 2018). The metabolite of *S. gordonii*, 4-aminobenzoate/p-aminobenzoic acid (pABA), can increase the subgingival colonization and intracellular survival of *P. gingivalis* but decrease its pathogenicity (Kuboniwa et al., 2017). Therefore, the interactions between bacterial species may affect bacterial colonization and pathogenicity. Although studies have proven that *F. nucleatum* can use the outer membrane proteins RadD and CmpA to adhere to *S. gordonii* and colonize the same ecological locus of the subgingival plaque (Kaplan et al., 2009; Lima et al., 2017), this largely remains to be investigated. Here, we evaluated the effects of coaggregation between *F. nucleatum* subsp. *polymorphum* and *S. gordonii* on the subgingival synergistic interactions to hGECs and analyzed the potential mechanisms in the development of periodontal disease.

The multilayer model of hGECs infected with *P. gingivalis*, *A. actinomycetemcomitans, F. nucleatum or S. gordonii* showed that *P. gingivalis* invaded intracellularly and spread cell to cell, *A. actinomycetemcomitans* and *F. nucleatum* remained extracellular and showed intercellular movement through the multilayer, while *S. gordonii* remained extracellular and predominantly associated with the superficial cell layer (Dickinson et al., 2011). Although the study established a multilayer structure of hGECs to stimulate the actual oral environment, oral bacteria usually did not infect hGECs in monocultures. The interactions between oral bacteria may influence adhesion to and invasion of hGECs. A previous study reported that *F. nucleatum* could promote noninvasive *Streptococcus cristae* and *Streptococcus sanguinis* adhesion to and invasion of hGECs (Edwards et al., 2006). Studies on the polymicrobial infections of hGECs showed that *F. nucleatum* improved the adhesion and invasion of periodontal pathogens *P. gingivalis* and *A. actinomycetemcomitans* in hGECs (Saito et al., 2009; Saito et al., 2012; Li et al., 2015). At the same time, *P. gingivalis* has been confirmed to inhibit *F. nucleatum* invasion of hGECs by gingipain when in a coinfection state (Jung et al., 2017). Current studies have mostly focused on monoculture infection or polymicrobial infection with periodontal pathogens, and the effects of the interactions between *F. nucleatum* and *S. gordonii* on the adhesion and invasion of hGECs remain to be investigated. In the present study, coinfection with *F. nucleatum* subsp. *polymorphum* improved *S. gordonii* adhesion to hGECs more significantly than coaggregation with *F. nucleatum* subsp. *polymorphum*, indicating that different mechanisms functioned during the two types of infections. As for *F. nucleatum* subsp. *polymorphum*, coaggregation with *S. gordonii* significantly inhibited the adhesion and invasion of hGECs. Based on a previous study reporting that *F. nucleatum* could survive in hGECs for no more than 12 h (Ji et al., 2010), it was speculated that coaggregation may improve the extracellular survival of *F. nucleatum* subsp. *polymorphum* by inhibiting its adhesion and invasion of hGECs.

In our study, infection time within 24 h was considered appropriate for evaluations because after 48 h of infection, the cell proliferation activity was significantly inhibited in both coaggregation and coinfection groups. Meanwhile, the cell apoptosis rate was significantly increased in *S. gordonii* monoculture, coaggregation, and coinfection groups after 48 h of infection, with the highest cell apoptosis rate nearly 80% in *S. gordonii* monoculture group. This may be related to the exhaustion of media nutrients caused by the accumulated amount of *S. gordonii* in these three groups, making it challenging for hGECs proliferation and survival. In the present study, *F. nucleatum* subsp. *polymorphum* significantly promoted hGECs apoptosis after 2 h and sustained through 24 h of infection (Dickinson et al., 2011), which was consistent with the previous study. However, a weak ability to induce cell apoptosis was found in *S. gordonii* before 12 h, possibly because of a symbiotic relationship between *S. gordonii* and hGECs in the early stage of infection. According to the previous study, *P. gingivalis* could activate the phosphoinositide 3-kinase (PI3K) signaling pathway to inhibit the apoptosis of hGECs when coinfected with *F. nucleatum*, facilitating the intracellular survival of *P. gingivalis* and *F. nucleatum* (Maekawa et al., 2014). This suggested that bacteria in monoculture or coinfection resulted in different regulatory mechanisms of cell apoptosis. In the present study, coaggregation of *F. nucleatum* subsp. *polymorphum* and *S. gordonii* showed similar effects on hGECs proliferation activity with coinfection, while coaggregation showed an inhibitory effect on cell apoptosis at 24 h of infection compared with coinfection. This suggested that, compared with coinfection of the two species, coaggregation inhibited hGECs apoptosis which may facilitate the intracellular survival of bacteria and favor a prolonged cell inflammation induction.

*F. nucleatum* and lipopolysaccharide can stimulate the secretion of proinflammatory cytokines and chemokines, leading to inflammation and bone resorption (Huang et al., 2004; Hasegawa et al., 2007; de Andrade et al., 2019; Kantrong et al., 2019). The outer membrane proteins FadA and Fap2 of *F. nucleatum* are involved in both bacterial coaggregation mediation and infection of various host cells which induce inflammatory responses (Xu et al., 2007; Ikegami et al., 2009; Fardini et al., 2011). Lipoteichoic acid and lipoprotein are the main virulence factors of *S. gordonii* in bacterial infection and inflammatory responses (Bi et al., 2017). Studies have shown that *F. nucleatum* significantly promotes the secretion of IL-6, IL-8, and IL-1β in hGECs, with no significant effect on IL-10 secretion, while *S. gordonii* has no significant effect on the secretion of IL-6, IL-8, IL-1β, and IL-10 at 4 h or 24 h of infection (Ji et al., 2007; Stathopoulou et al., 2010) and even inhibits IL-6 and IL-8 secretion during 8 h of infection (Hasegawa et al., 2007). Another study showed that *F. nucleatum* significantly promoted the secretion of TNF-α and IL-1β, while *S. gordonii* promoted the secretion of TNF-α, IL-6, IL-8, and IL-1β after 24 h of infection (Dickinson et al., 2011). The inconsistency of the results may be because of the different MOIs, bacterial subspecies, or epithelial cell models (monolayer or multilayers). Most studies *in vitro* investigated the secretion levels of inflammatory cytokines by bacterial stimuli were within 24 h of infection. In the present study, we found that the secretion levels of inflammatory cytokines in each group had a relatively consistent trend of variety from 0.5 h to 24 h (**Figure 9**). However, not all groups maintained the original trend after 48 h, especially for IL-6 secretion level in the coinfection group, which was dramatically increased and higher than the coaggregation group after 48 h of infection. The precise reason for this fluctuation in IL-6 secretion level was not clear. However, during the experiment, we observed that the floating debris or dead cells were much more obvious in the three groups infected with *S. gordonii* monoculture, coinfection and coaggregation of *F. nucleatum* subsp. *polymorphum* and *S. gordonii* after 48 h of infection. Combined with the significant decrease in cell proliferation activity and significant increase in cell apoptosis after 48 h in the present study, these results may all be related to the exhaustion of media nutrients caused by the accumulated amount of *S. gordonii* in these three groups. Therefore, in the present study, we focused on analyzing and concluding the changes of secretion levels of inflammatory cytokines among the groups within 24 h of infection. Nevertheless, the results showed that compared with coinfection, coaggregation between *F. nucleatum* subsp. *polymorphum* and *S. gordonii* promoted hGECs to secrete the proinflammatory factors TNF-α and IL-6 at 24 h of infection, while inhibiting the secretion of the anti-inflammatory factor TGF-β1. Different from studies *in vitro*, animal studies usually took a long-term evaluation of host responses (Polak et al., 2009; Polak et al., 2012; de Molon et al., 2014). An animal study showed that at 42 days post-infection, coinfection with *F. nucleatum* and *P. gingivalis* synergistically promoted the loss of periodontal bone tissue and aggravated inflammatory responses in rats (Polak et al., 2009). Animal experiments with long-term evaluations could be used to explore and verify the specific mechanisms for further study.

It was reported that the NF-κB and MAPK signaling pathways were involved in IL-8 secretion by hGECs infected with *F. nucleatum* (Huang et al., 2004). TLR2 and TLR4 simultaneously mediated the secretion of IL-6 and TNF-α by hGECs infected by *F. nucleatum*, which also activated the NF-κB and MAPK signaling pathways (Park et al., 2014). Compared with hGECs infected by *F. nucleatum* monoculture, coinfection with *P. gingivalis* or *A. actinomycetemcomitans* significantly reduced the secretion of IL-8 and inhibited host inflammatory responses after 4 h of infection (Li et al., 2015). In the present study, western blot results verified significant activation of NF-κB in the coaggregation group at 0.5 h and 2 h, as well as significant activation of MAPK at 6 h and 12 h of infection. This indicated that both NF-κB and MAPK signaling pathways were involved in the regulatory effect of coaggregation of the two species on inflammatory responses, with NF-κB activation at an earlier stage of infection. However, an absence of an effect on IL-8 was observed based on the phosphorylation of p65. Although IL-8 is a classical downstream of the NF-κB signaling pathway, the regulatory mechanism of IL-8 seems to be complex. A previous study showed MK2 was involved in regulating the TNF-induced expression of IL-8 by p38 MAPK in human lung microvascular endothelial cells at a post-transcriptional level (Su et al., 2008). Another study showed the stimulation of synovial fibroblasts with IL-6 and TNF-α cooperatively inhibited the induction of IL-8 (Valin et al., 2020). It was speculated that a more complex mechanism in IL-8 secretion existed induced by coaggregation of *F. nucleatum subsp. polymorphum* and *S. gordonii*.

In the present study, no significant changes in *TLR4* gene expression levels were observed in hGECs infected by *F. nucleatum* subsp. *polymorphum* monoculture. This may be because a relatively lower MOI was used than previous studies in which the MOI was 200 or 1000 (Ji et al., 2009; Sun et al., 2010). The MOI was limited to 100 in the present study because the number of *S. gordonii* was the same as that of *F. nucleatum* subsp. *polymorphum* for coaggregation. *S. gordonii* at a larger MOI grew exponentially and caused cell apoptosis or death rapidly because of the accelerated consumption of nutrients. At the transcriptional level, the coaggregation of *F. nucleatum* subsp. *polymorphum* and *S. gordonii* upregulated the expression levels of *TLR2* and *TLR4* in hGECs, but the expression levels were lower than those in hGECs infected by coinfection of the two species. This may indicate that the activation of the NF-κB and MAPK signaling pathways in hGECs infected by coaggregation of the two species did not occur through the upregulation of *TLR2* and *TLR4*, but through the enhanced bacterial virulence induced by coaggregation. Moreover, compared with hGECs infected with coinfection bacteria, coaggregation inhibited the secretion of the anti-inflammatory cytokine TGF-β1, suggesting that coaggregation of *F. nucleatum* subsp. *polymorphum* and *S. gordonii* could aggravate the cellular inflammatory response through a two-way regulation of proinflammatory and anti-inflammatory cytokines. Interestingly, the secretion of TGF-β1 did not change with the use of related pathway antagonists/inhibitors. The underlying mechanism of the regulation of TGF-β1 secretion in hGECs induced by coaggregation still needs to be investigated.

By RNA-Seq, Mutha et al. (Mutha et al., 2018) found that by comparison with monocultures, 16 genes were regulated following coaggregation in *F. nucleatum* subsp. *nucleatum* whereas 119 genes were regulated in *S. gordonii*. In both species, genes involved in amino acid and carbohydrate metabolism were strongly affected by coaggregation (Mutha et al., 2018). Our previous transcriptome results indicated up-regulated genes associated with protein export systems and repressed arginine biosynthesis in *S. gordonii* after coaggregation might help enhance and maintain a symbiotic relationship with *F. nucleatum* subsp. *polymorphum* (Liu et al., 2021). In *F. nucleatum* subsp. *polymorphum*, genes related to LPS or peptidoglycan biosynthesis were downregulated, which might reduce the immunogenicity of *F. nucleatum* subsp. *polymorphum* and improve bacterial survival within macrophages (Liu et al., 2021). Besides, the coaggregation of *F. nucleatum* subsp. *polymorphum* and *S. gordonii* exhibited significantly decreased levels of propanoic acid and butyric acid than dual-species co-cultures (Liu et al., 2021). The symbiotic lifestyle and metabolic changes of *F. nucleatum* subsp. *polymorphum* and *S. gordonii* after dual-species coaggregation may contribute to the regulatory effect on the synergistic virulence to hGECs in the present study. In further study, bacterial mutants should be constructed for more rigorous conclusions and validations.

In contrast to previous studies that only considered *S. gordonii* as an early colonizer, our study revealed that the functions of *S. gordonii* coaggregated with *F. nucleatum* subsp. *polymorphum* in the periodontal virulence. The regulatory effect of interactions between *F. nucleatum* subsp. *polymorphum* with *S. gordonii* in the process of periodontal diseases may be more fully interpreted. In further studies, animal models of the colonization of coaggregated bacteria on the tooth surface or gingival sulcus are needed to investigate of the potential mechanism.

## Conclusions

In summary, the coaggregation between *F. nucleatum* subsp. *polymorphum* and *S. gordonii* inhibited the adhesion and invasion of *F. nucleatum* subsp. *polymorphum* to hGECs but enhanced the adhesion of *S. gordonii* to hGECs. Coaggregation between *F. nucleatum* subsp. *polymorphum* and *S. gordonii* coordinately promoted the secretion of the proinflammatory cytokines TNF-α and IL-6 by hGECs through the TLR/NF-κB and TLR/MAPK signaling pathways, while inhibiting the secretion of the anti-inflammatory cytokine TGF-β1, thus aggravating the inflammatory response of hGECs.

## Data Availability Statement

The original contributions presented in the study are included in the article/**Supplementary Material**. Further inquiries can be directed to the corresponding authors.

## Author Contributions

LG and RY conceived, designed, and performed experiments. XW, LG, and RY analyzed the data. RY wrote the manuscript. XW, LG, and ZL reviewed and edited the manuscript. All authors contributed to the article and approved the submitted version.

## Funding

This work was supported by the National Natural Science Foundation of China (grant number: 81670982). The funders did not play a role in manuscript design, data collection, data analysis, data interpretation, or writing of the manuscript.

## Conflict of Interest

The authors declare that the research was conducted in the absence of any commercial or financial relationships that could be construed as a potential conflict of interest.

## Publisher’s Note

All claims expressed in this article are solely those of the authors and do not necessarily represent those of their affiliated organizations, or those of the publisher, the editors and the reviewers. Any product that may be evaluated in this article, or claim that may be made by its manufacturer, is not guaranteed or endorsed by the publisher.

## Supplementary Material

Supplementary Figure 1
**(A)** Extracellular bacteria after antibiotic treatment with 200 μg/mL metronidazole and 300 μg/mL gentamicin for 60, 90 and 120 min (***p <0.01, ***p <0.001*). **(B)** The effect of antibiotic treatment on hGECs proliferation activity for 60, 90 and 120 min.

Supplementary Figure 2 Flow cytometry images of hGECs infected by Fnp, Sg, Fnp+Sg and Fnp-Sg after various time points.
