# Supplementary figures and images for: The Regulatory Effect of Coaggregation Between Fusobacterium nucleatum and Streptococcus gordonii on the Synergistic Virulence to Human Gingival Epithelial Cells
Source: Front Cell Infect Microbiol. 2022 Apr 29;12:879423. doi: 10.3389/fcimb.2022.879423 (PMC9100429; doi:10.3389/fcimb.2022.879423)

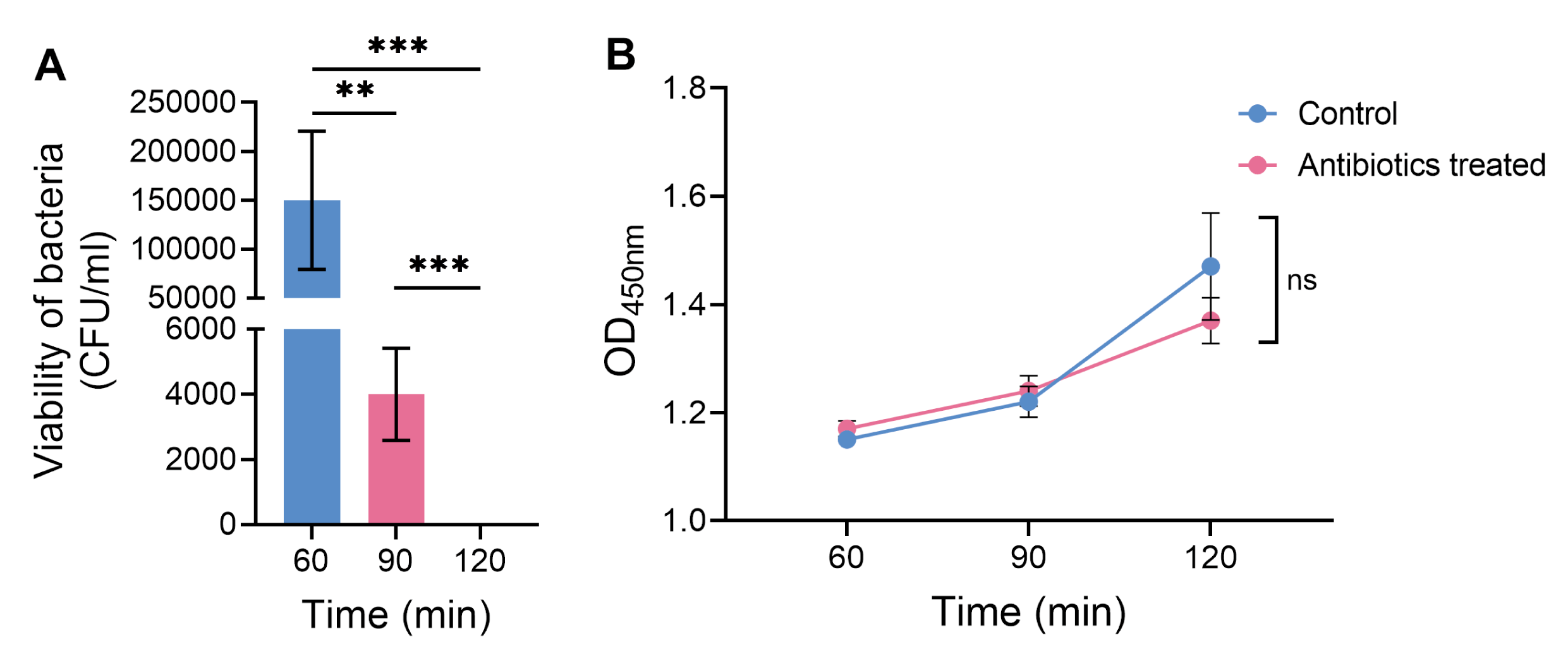

Supplement: Supplementary Figure 1 — (A) Extracellular bacteria after antibiotic treatment with 200 μg/mL metronidazole and 300 μg/mL gentamicin for 60, 90 and 120 min (**p <0.01, ***p <0.001). (B) The effect of antibiotic treatment on hGECs proliferation activity for 60, 90 and 120 min. [file Image_1.tif]

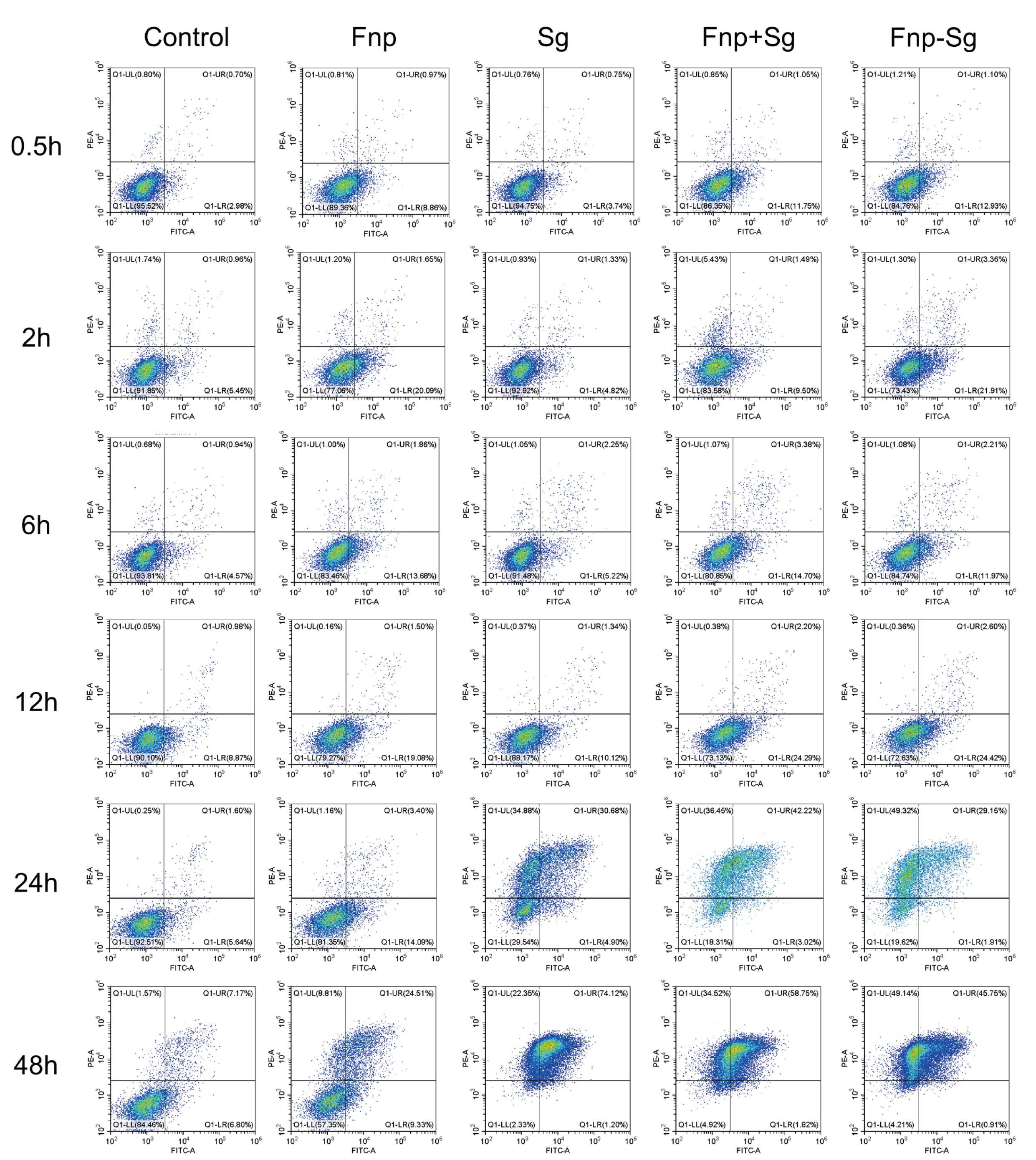

Supplement: Supplementary Figure 2 — Flow cytometry images of hGECs infected by Fnp, Sg, Fnp+Sg and Fnp-Sg after various time points. [file Image_2.tif]
